# Financial ties between leaders of influential US professional medical associations and industry: cross sectional study

**DOI:** 10.1136/bmj.m1505

**Published:** 2020-05-27

**Authors:** Ray Moynihan, Loai Albarqouni, Conrad Nangla, Adam G Dunn, Joel Lexchin, Lisa Bero

**Affiliations:** 1 Institute for Evidence-Based Healthcare, Bond University, Gold Coast, QLD, 4229, Australia; 2 Discipline of Biomedical Informatics and Digital Health, The University of Sydney, NSW, Australia; 3 School of Health Policy and Management, York University, Toronto, Canada; 4 Charles Perkins Centre and School of Pharmacy, Faculty of Medicine and Health, The University of Sydney, NSW, Australia

## Abstract

**Objective:**

To investigate the nature and extent of financial relationships between leaders of influential professional medical associations in the United States and pharmaceutical and device companies.

**Design:**

Cross sectional study.

**Setting:**

Professional associations for the 10 costliest disease areas in the US according to the US Agency for Healthcare Research and Quality. Financial data for association leadership, 2017-19, were obtained from the Open Payments database.

**Population:**

328 leaders, such as board members, of 10 professional medical associations: American College of Cardiology, Orthopaedic Trauma Association, American Psychiatric Association, Endocrine Society, American College of Rheumatology, American Society of Clinical Oncology, American Thoracic Society, North American Spine Society, Infectious Diseases Society of America, and American College of Physicians.

**Main outcome measures:**

Proportion of leaders with financial ties to industry in the year of leadership, the four years before and the year after board membership, and the nature and extent of these financial relationships.

**Results:**

235 of 328 leaders (72%) had financial ties to industry. Among 293 leaders who were medical doctors or doctors of osteopathy, 235 (80%) had ties. Total payments for 2017-19 leadership were almost $130m (£103m; €119m), with a median amount for each leader of $31 805 (interquartile range $1157 to $254 272). General payments, including those for consultancy and hospitality, were $24.8m and research payments were $104.6m—predominantly payments to academic institutions with association leaders named as principle investigators. Variation was great among the associations: median amounts varied from $212 for the American Psychiatric Association leaders to $518 000 for the American Society of Clinical Oncology.

**Conclusions:**

Financial relationships between the leaders of influential US professional medical associations and industry are extensive, although with variation among the associations. The quantum of payments raises questions about independence and integrity, adding weight to calls for policy reform.

## Introduction

A growing body of evidence details the nature and extent of financial associations between health professionals and the pharmaceutical and device industries, and the impacts of that involvement on research integrity, medical education, and patient care.^1^ ^2^ ^3^ ^4^ Studies have found that sponsored trials have more favourable outcomes for sponsors’ products,^5^ sponsored education is associated with higher prescribing of sponsors’ medicines,^6^ and guideline panels, which change and often expand disease definitions, are populated by doctors with extensive financial conflicts of interest.^7^ In response to concerns about this evidence, the creation of mandated transparency databases such as the United States Sunshine Act’s Open Payments system now enable more enhanced investigation of these financial relationships. Despite their influence over key aspects of medicine,^8^ the leaders of professional medical associations have received limited scrutiny about their relationships with industry.

Professional medical associations such as doctor’s colleges and societies play vitally important roles within healthcare systems. They represent health professionals, fund research, facilitate medical education, and produce guidelines that influence practice and set disease definitions. As one author observed, the influence of professional medical associations is “so wide-ranging, involving almost all aspects of medicine, that scientific integrity, objectivity, and independence are essential.”^8^ Although debate is ongoing about how close the relationship between professional medical associations and industry should be,^8^ ^9^ ^10^ there are a paucity of data on the relationships between industry and the leaders of these associations.^11^ We undertook an analysis of the leadership across a sample of influential US professional medical associations, using the US government’s Open Payments database. Since 2013, this database has disclosed payments and transfers of value (collectively referred to as payments hereafter) to US based medical doctors and doctors of osteopathy from drug and device makers, across multiple categories, including research payments and general payments for consultancy, royalties, and hospitality.^12^

As concern and evidence about relationships between industry and professionals have grown, the integrally related problem of overuse of medical tests, diagnoses, and treatments has also attracted increasing attention. Estimates in the US suggest that at least 20% of healthcare spending might be wasted, including on overtreatment,^13^ and a report by the Organisation for Economic Co-operation and Development on waste similarly estimates that 20% of health spending could be directed towards better use.^14^ In response, initiatives such as Choosing Wisely have emerged, and leading journals have launched campaigns such as *Too Much Medicine*, and *Less is More*, which includes annual systematic reviews of literature on medical overuse.^15^ A recent analysis identified commercial factors as one set of drivers of overdiagnosis, overuse, and overmedicalisation.^16^ As efforts to deal with the problem of too much medicine intensify, relationships between health professionals and industries interested in maximising markets are attracting more scrutiny.^2^ In this study we identified and analysed the nature and extent of financial relationships between the leaders of a sample of influential professional medical associations in the US and drug and device makers. A secondary aim was to identify the extent to which recent guidelines from these associations mentioned concerns about overdiagnosis or overuse.

## Methods

As per our protocol (see supplementary file) we conducted a cross sectional study of the financial relationships between pharmaceutical and device manufacturers and the leaders of influential US professional medical associations active across common costly disease areas.

### Identifying disease areas and professional medical associations

We used the most recent US Agency for Healthcare Research and Quality (2015) data to identify the top 10 costliest disease areas in the US,^17^ based on a belief that this number would offer an important range of conditions. The costliest represent large disease burdens, such as cancer, and include areas with concerns about the potential existence of overdiagnosis and overuse.^15^

To identify a leading professional medical association—one that represents doctors and produces guidelines—for each of the 10 disease areas, we sought recommendations from three US based peers in each of the 10 disease areas. We identified professional medical associations led predominantly by doctors for doctors, rather than broader civil society organisations active within specific disease areas. Members of the authorship team identified peers and included those drawn from members of Cochrane Review Groups working in the relevant diseases and from an independent expert list (https://jeannelenzer.com/list-independent-experts). An example of the request sent to expert peers is included in the supplementary file.

### Identifying association leaders and those in Open Payments

Using published materials, including association websites and annual reports, two authors (RM, LA) independently identified the leaders of each identified medical association. Any discrepancies were resolved by discussion. Leaders were defined as members of the association’s overarching body or predominant leadership entity, such as a member of the board or governing council but not of subcommittees. We included members for the current year of service (2019) and two years previously.

For each included leader, two authors (RM, LA) then independently identified whether he or she appeared in the Open Payments database, which includes payments to medical doctors and doctors of osteopathy but does not include payment to other professionals, such as nurses. To ensure a correct match between the leader and the person identified in Open Payments, biographical information, such as name and affiliation, was compared. Discrepancies were resolved by discussion, and a unique URL link to Open Payments was prepared for each leader who appeared in that database. We also noted whether the association included publicly available financial disclosure information about leadership, together with the current listings of the leaders’ names.

### Identifying and extracting payment details and companies

In line with World Health Organization and other guidance on relevant conflicts of interest,^1^ ^18^ we identified any financial relationships an individual might have had in their current year of board membership (if membership was 2017-18, the current year was identified as 2017) and the four years before and one year after membership. By using the unique link for each leader identified by two authors independently, one author (CN) manually extracted data on payments (from April to September 2019) for the identified leaders who appeared in Open Payments, using categories within the database: chiefly general payments, research, associated research, and others, such as investments. General payments include but are not limited to consultancy, royalties, and hospitality. Research payments include those where the company making the payment names the individual doctor as primary recipient, and payments to institutions, where the doctor is named as a principal investigator on the research. We also downloaded the data from Open Payments directly into Excel spreadsheets, which included the names of the companies making payments. A second author (RM) double checked the information from Open Payments for accuracy.

### Identifying guidelines and mentions of overdiagnosis and overuse

For each association, two authors (RM, LA) independently identified up to three guidelines related to the relevant disease area. We identified guidelines with the highest combination of downloads (using journal website metrics) and citations (using Web of Science) for each of the three most recent years, when available. When no guidelines were available for a particular year, we chose the next most recent year. Discrepancies and uncertainties in identifying guidelines, including uncertainty about which guideline had the highest combination of citations and downloads, were resolved by discussion. We used a simple method to identify mentions of overdiagnosis and overuse in the guidelines, drawing on methods previously used by authors.^7^ Two authors (RM, plus one other author) independently identified whether mentions were explicit or used related terms, or were implicit, and whether any guideline recommendations had resulted from those mentions. This process was piloted with all authors using one guideline. Any discrepancies were resolved by discussion or referred to a third author.

### Outcome measures

We investigated several primary outcomes. Firstly, the proportion of leaders with financial ties to industry in their current year of tenure, the previous four years, and the year after, contingent on data availability in Open Payments, in which the most recent year of disclosed payments was 2018. Secondly, the overall proportion of US based leaders who were medical doctors or doctors of osteopathy with financial ties, and the proportion within each organisation. Thirdly, the proportion of associations with no leaders with financial ties. Fourthly, among leaders with ties, the amount of payments (in dollars) overall and within three categories (general, research and associated research combined, other). Fifthly, the total amounts overall and for each association and each leader. Finally, the top three contributor companies (in dollar amounts) to each association’s leaders. A secondary outcome included the proportion of the three most widely used guidelines (by association for the target disease areas) explicitly or implicitly mentioning overdiagnosis or overuse, or both, and related issues.

### Changes to protocol

Our protocol included a plan to investigate any changes in the extent or nature of financial ties over the study period, which we decided not to pursue given the short three year window. Our protocol did not include a plan to assess leaders by sex, but after noting large sex imbalances between some association leaders, we decided to include these data.

### Patient and public involvement

There was no patient or public involvement in developing the research question, the analysis, or manuscript preparation for this study. An approach to a health consumer organisation seeking potential involvement in developing and running the research was unsuccessful.

## Results

The 10 costliest disease areas in the US were heart disease, trauma related disorders, mental disorders, diabetes mellitus, osteoarthritis and other non-traumatic joint disorders, cancer, chronic obstructive pulmonary disease and asthma, back problems, infectious diseases, and hypertension. Table 1 lists the identified professional medical associations. No association was clearly identified for hypertension. However, as some expert peers had suggested the American College of Physicians for other conditions, including back problems, diabetes, and for hypertension, it was decided to include this association in the list of 10 associations. 

**Table 1:** Ten US professional medical associations across 10 costliest disease areas, and payments ($) to leaders from pharmaceutical and device companies

Association	No of leaders (% with ties)	No of women leaders (% with ties)	Total payments			General payments			Research payments			Other payments	
			Total	Median (interquartile range) for those with ties		Total	Median (interquartile range) for those with ties		Total	Median (interquartile range) for those with ties		Total	Median (interquartile range) for those with ties
American College of Cardiology, ACC	26 (88)	5 (60)	22 974 724	49 143 (1654 to 339 509)		2 019 702	21 867 (847 to 49 657)		20 955 022	25 747 (0 to 240 858)		0	0 (0 to 0)
American College of Physicians, ACP	41 (61)	15 (53)	569 096	404 (82 to 5344)		401 370	319 (82 to 2593)		167 725	0 (0 to 0)		0	0 (0 to 0)
American College of Rheumatology, ACR	33 (88)	15 (73)	17 671 146	251 464 (3226 to 960 624)		1 251 469	5191 (726 to 56 921)		16 397 177	147 797 (0 to 903 703)		0	0 (0 to 0)
American Psychiatric Association, APA	43 (37)	20 (30)	344 745	212 (87 to 3596)		128 737	163 (74 to 1314)		216 008	0 (0 to 0)		0	0 (0 to 0)
American Society of Clinical Oncology, ASCO	30 (80)	8 (100)	55 581 907	518 092 (136 419 to 1 958 745)		1 464 771	21 138 (10 548 to 89 399)		54 117 136	510 746 (37 237 to 1 830 666)		0	0 (0 to 0)
American Thoracic Society, ATS	51 (69)	26 (58)	3 581 684	6468 (689 to 99 265)		2 230 531	4426 (148 to 17 709)		1 351 153	0 (0 to 40 558)		0	0 (0 to 0)
Endocrine Society, ES	29 (66)	16 (63)	7 125 517	44 383 (9110 to 130 643)		1 989 181	15 095 (1506 to 32 099)		5 136 336	4629 (0 to 115 718)		0	0 (0 to 0)
Infectious Diseases Society of America, IDSA	29 (93)	14 (100)	5 950 053	31 805 (3682 to 207 540)		1 016 905	6419 (2078 to 44 237)		4 933 148	0 (0 to 139 958)		0	0 (0 to 0)
North American Spine Society, NASS	32 (75)	2 (0)	10 705 645	24 521 (2014 to 338 386)		9 503 666	7948 (820 to 265 602)		1 033 607	0 (0 to 9363)		168 372	0 (0 to 0)
Orthopaedic Trauma Association, OTA	14 (93)	1 (100)	5 319 783	87 151 (26 212 to 413 890)		4 736 517	68 224 (13 774 to 156 325)		286 572	0 (0 to 0)		296 694	0 (0 to 0)
All Associations	328 (72)	122 (62)	129 824 300	31 805 (1157 to 254 272)		24 742 850	6026 (309 to 54 167)		104 593 885	0 (0 to 132 913)		487 566	0 (0 to 0)

£1 (£0.80; €0.90).

An extreme outlier was excluded from analysis (total payments for an individual of $240m). For three associations data could not be found for one year of leadership: North American Spine Society, Orthopaedic Trauma Association, and Endocrine Society. Zero amounts do not imply missing data, rather that the specific category had no payments. According to the Open Payments website “Research payments can include direct compensation to physicians, funding for research study coordination and implementation, or payments to study participants to cover expenses associated with the study” and “The physician research payments total includes: 1.) Payments where the company making the payment has named a physician as the primary recipient, and 2.) Payments to a research institution or entity where a physician is named as a principal investigator on the research project (i.e. received associated research funding).”

Overall, 328 leaders were identified across the three most recent years of board membership of the associations. Using the Open Payments database, 235 (72%) were found to have had any financial ties to industry within the year of membership, the four years before membership, and the year after membership. Of the 293 leaders who were medical doctors or doctors of osteopathy (only three), 235 (80%) had any financial ties to industry. No organisation had a leadership free of financial ties.

**Fig 1:**
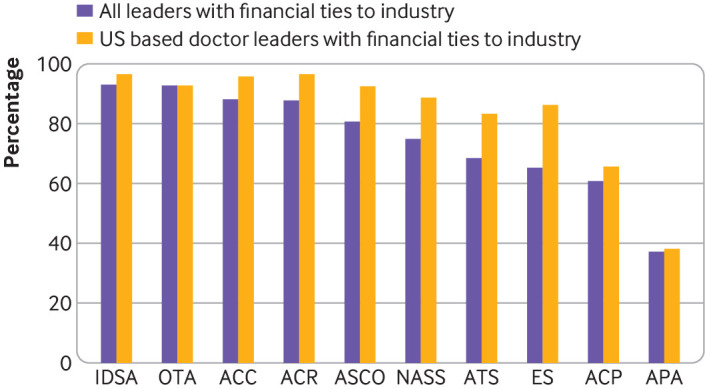
Proportion of leaders of professional medical associations with financial ties to industry. IDSA=Infectious Diseases Society of America; OTA=Orthopaedic Trauma Association; ACC=American College of Cardiology; ACR=American College of Rheumatology; ASCO=American Society of Clinical Oncology; NASS=North American Spine Society; ATS=American Thoracic Society; ES=Endocrine Society; ACP=American College of Physicians; APA=American Psychiatric Association

Table 1 shows the proportions of leaders with financial ties by association and payment category. Although for most associations more than 80% of their US based medical leaders had financial ties, the American College of Physicians had 66% and the American Psychiatric Association had 38% (fig 1).

Total payments of almost $129.9m ($130m, £103m; €119m) (median $31 805, interquartile range $1157 to $254 272) were linked to the 235 leaders with financial ties (table 1). That total included almost $24.8m for general payments ($6026, $309 to $54 167), $104.6m for research ($0, $0 to $132 913), predominantly to institutions with leaders named as principle investigators, and $0.5m ($0, $0 to $0) for other payments.

**Fig 2:**
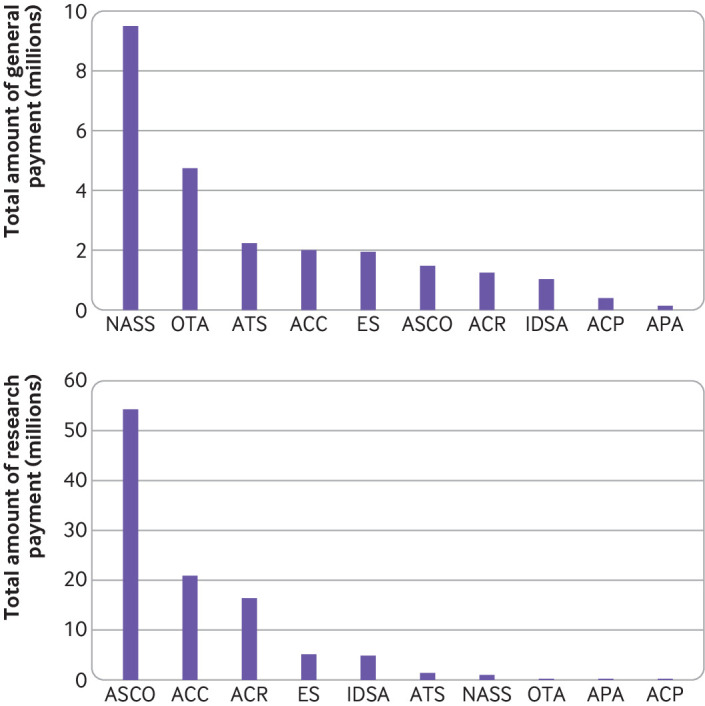
General and research payments to leaders of 10 professional medical associations. General payments include for consultancies, travel, food, and beverages, and royalties. IDSA=Infectious Diseases Society of America; OTA=Orthopaedic Trauma Association; ACC=American College of Cardiology; ACR=American College of Rheumatology; ASCO=American Society of Clinical Oncology; NASS=North American Spine Society; ATS=American Thoracic Society; ES=Endocrine Society; ACP=American College of Physicians; APA=American Psychiatric Association

The amounts of general payments and research payments varied widely (fig 2). Leaders of the North American Spine Society received more than $9.5m for general payments and those of the Orthopaedic Trauma Association received more than $4.7m, whereas leaders of the American College of Physicians received just over $400 000 and those of the American Psychiatric Association received around $129 000. Research payments linked to leaders of the American Society of Clinical Oncology were over $54m and for those of the American College of Cardiology, almost $21m, whereas for leaders of the American Psychiatric Association the figure was just over $216 000 and for those of the American College of Physicians just under $168 000 (fig 2).

**Fig 3:**
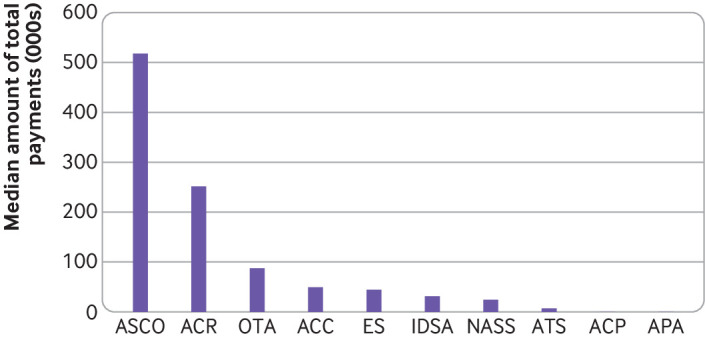
Median payment amounts to leaders of professional medical associations with ties to industry. IDSA=Infectious Diseases Society of America; OTA=Orthopaedic Trauma Association; ACC=American College of Cardiology; ACR=American College of Rheumatology; ASCO=American Society of Clinical Oncology; NASS=North American Spine Society; ATS=American Thoracic Society; ES=Endocrine Society; ACP=American College of Physicians; APA=American Psychiatric Association

Analysis of median total amounts linked to individual leaders also varied widely between associations (fig 3). The median was $518 000 for leaders of the American Society of Clinical Oncology and more than $251 000 for those of the American College of Rheumatology compared with just $404 for leaders of the American College of Physicians and $212 for those of the American Psychiatric Association. Box 1 shows three examples of financial relationships between three leaders of professional medical associations and industry, reflecting the upper quartile, the median, and the lower quartile of payments for these three associations.

Box 1 **Examples of financial relationships between three leaders of professional medical associations and industry**

**American Society of Clinical Oncology board member**

$2.4m over six years, including general payments of $441 000, including $97 000 in one year for “compensation for services . . . including serving as faculty or as a speaker”

**Endocrine Society council member**

$44 000 over five years, including general payments of $40 000, including a $8500 consulting fee in one year

**American College of Physicians board member**

$147 over four years, including $18 for food and beverages

Supplementary table 1 shows the top three companies providing payments for each associations’ leadership and the amount of payments, ranging from almost $17m from Astra Zeneca to less than $30 000 from Shire. Only two of the 10 associations had publicly available information about the relationships of leaders with industry accompanying their leadership list: American College of Rheumatology and American Society of Clinical Oncology. An unplanned analysis of leaders by sex showed that women comprised 122 of 328 leaders (37%), ranging from 6% of the board of the North American Spine Society to 55% of the leadership of the Endocrine Society. Overdiagnosis, overuse, or associated problems were explicitly mentioned in six of 28 guidelines, implicitly mentioned in a further four, and these mentions were reflected in recommendations in seven (supplementary table 1).

## Discussion

Almost three quarters of the leaders of 10 influential professional medical associations in the US, representing and educating doctors working across the most common and costliest disease areas, had financial relationships with pharmaceutical and device manufacturers. Based on analysis of publicly available payment data for the year of service as a leader and the four years before and one year after membership, leaders of the associations were linked to payments totalling almost $130m from industry. The median amount for each leader across this timeframe was more than $31 000. Results also indicate strong variation in the amount of payments among associations, with median amounts for each leader varying from $212 for the American Psychiatric Association to $518 000 for the American Society of Clinical Oncology. The largest research payments flowed to leaders of the American Society of Clinical Oncology ($54m) and the American College of Cardiology ($21m). The largest general payments—which can include fees for consultancy, speaking, royalties, and other payments—were given to leaders of the North American Spine Society ($9.5m) and the Orthopaedic Trauma Association ($4.7m).

### Limitations and strengths of this study

This study has important limitations. We relied solely on the US government’s Open Payments database, although arguably this is the most comprehensive and reliable source for financial payments. Without any objective list available, identifying associations to match the 10 costliest disease areas was mostly straightforward, although in some cases uncertain, and we make no claim that the 10 associations identified are the definitive list of the most influential. We explicitly excluded not-for-profit civil society organisations with broader remits, such as the American Heart Association and American Diabetes Association, focusing instead on associations led predominantly by medical doctors for doctors, which limits generalisability of findings. Owing to data availability, a complete set of payments is not yet available for leaders in 2018 and 2019, suggesting our results underestimate the total amount of payments. In three cases we were unable to find a leadership list for an association for a specific year, further adding to underestimation. And finally, our simple checking of guidelines for any mention of overdiagnosis or overuse made no judgments about guideline quality and was merely using a surrogate marker for the extent to which popular guidelines are dealing with these health challenges. Notwithstanding these limitations, this study investigated the extensive financial relationships between the leaders of a sample of influential US professional medical associations and industry, derived from an objective list of a broad range of disease areas and subjective practical expert advice.

### Comparison with other studies

Currently, few data exist on financial ties of leaders of professional medical associations. One similar although limited study of leaders from Japan in 2016, where payments for research were not then available, found that 87% of board members of 19 medical associations received payments from industry totalling $6.5m.^11^ Other recent related research found that in 2014, 51% of US medical journal editors received general payments and 20% received research payments,^19^ with the highest median general payments in endocrinology ($7207, interquartile range $0 to $85 816) and cardiology ($2664; $0 to $12 912), whereas 87% of US gastroenterologists received industry payments in 2016 totalling more than $67m.^20^

### Implications of this study

In our study, the extent of involvement between industry and leaders of influential professional medical associations adds weight to calls for more independence.^1^ As one author observed, given their essential role in “maintaining and promoting the quality of medical care” and in order to show “independence and integrity,” leaders of professional medical associations must be “free of all financial ties with industry,” which he argued is feasible.^8^ As others have observed, guidelines from these professional medical associations “frequently call for greater use of health care services,” 21 and financial independence from commercial interests is doubly desirable if we are to tackle the problems of overuse and overdiagnosis.^22^ Although association board members rarely write their association guidelines, their leadership and influence guide the tone and approach for the entirety of their association’s work. Importantly, our study found the leadership of some professional medical associations, such as the American Psychiatric Association and American College of Physicians, to have significant numbers of members without ties, and those with ties generally only received negligible payments. This shows that financial independence from industry is possible for these medical leaders, and that in many cases payments are so low they could easily be phased out entirely, with simple policy reform within associations.

### Unanswered questions and conclusions

Major research questions remain unanswered about the financial relationships between professional medical associations and industry, the impacts on patient care, and the practicalities and potential value of reform to forge independence. Future research might investigate professional associations beyond the 10 studied here, and outside the US setting, and examine potential differences between primary care based groups and other specialties. With the advent of mandatory Sunshine Act style Open Payments databases in other nations, these relations could be tracked over time to help chart moves towards independence. Urgently needed are more detailed investigations of potential relationships between industry payments to associations, their leaders and guideline writers, and the nature of their guidelines, medical education,^23^ and advocacy on issues relevant to sponsors. Clearly, although there are growing calls for professional medical associations to sever ties with industry,^24^ nothing can or should compel them to do so. Conversely, as evidence on the extent of their ties grows, nothing should compel acceptance of their claims of independence or integrity. We support recommendations that doctors’ groups and their guideline writers become free of financial relationships with industry.^24^ Our study’s novel findings of enormous variation in the extent of these ties suggest that for some groups such independence will require time and major reform, whereas for others it will be quick and relatively easy.

**What is already known on this topic**

Evidence exists of extensive financial ties between health professionals and industry across many domains of healthcare
Calls for more independence from industry in production and use of evidence are growing
Few data are available on financial ties between industry and leadership of professional medical associations that are influential across research, education, and practice, including guideline development

**What this study adds**

During 2017-19, leaders of 10 influential US professional medical associations received almost $130m (£103m; €119m) from industry during their year of leadership, the four years before, and the year after
80% of the US based medical doctors who lead influential professional medical associations had financial relationships with industry, with great variation in the size of median payments by association, ranging from $212 to $518 000
Against a backdrop of growing calls for financial independence from commercial interests, these findings show that for some doctors’ groups this will require major reform, whereas for others it will be relatively easy

## Web extra

Extra material supplied by authors

**Supplementary information:** additional tables 1 and 2 and list of guidelines

**Supplementary information:** study protocol

**Supplementary information:** email sent to expert peers

## Notes

Ethical approval: This study was approved by the Bond University human research ethics committee (RM03089), and we stipulated that while working with publicly accessible data we would not name or describe any individual people in our results.

Data sharing: Deidentified simple summaries of data will be shared when possible and within confines of protocol and ethical approval conditions.

Dissemination to participants and related patient and public communities: We do not plan any participant dissemination as there were no participants and all data are already publicly available. We will widely disseminate the results of this study to professional colleagues and to the public, through conference and meeting presentations and media reporting.

The lead author (RM) affirms that the manuscript is an honest, accurate, and transparent account of the study being reported; that no important aspects of the study have been omitted; and that any discrepancies from the study as originally planned (and, if relevant, registered) have been explained.
