# Supplementary material for: Financial ties between leaders of influential US professional medical associations and industry: cross sectional study
Source: BMJ. 2020 May 27;369:m1505. doi: 10.1136/bmj.m1505 (PMC7251422; doi:10.1136/bmj.m1505)
Supplement: Supplementary file 1 — Supplementary information: additional tables 1 and 2 and list of guidelines [file moyr054932.wt1.pdf]

Table 1 Top three companies in dollar amounts to leadership of ten medical associations.

Association	Top 3 companies	Amount to leadership
ASCO	E.R. Squibb & Sons	$11,960,247
	Pfizer	$10,116,433
	Genentech	$5,018,152
ACC	Astra Zeneca	$16,824,864
	Edwards Lifesciences Corporation	$1,689,728
	Abbott Laboratories	$548,212
ACR	Janssen	$4,190,896
	E.R. Squibb & Sons	$4,118,202
	Pfizer	$1,938,326
NASS	Zimmer	$3,966,107
	Biomet	$2,739,106
	Stryker Corporation	$1,480,338
ES	Sanofi	$2,371,526
	Chugai Pharmaceutical	$1,501,960
	Novo Nordisk	$1,091,383
IDSA	Gilead Sciences	$1,756,568
	Merck Sharp & Dohme	$886,726
	Janssen	$399,950
OTA	Smith & Nephew Inc	$2,995,974
	Microport Orthopedics	$467,942
	Stryker Corporation	$385,988
ATS	Boehringer Ingelheim	$914,826
	AstraZeneca	$679,081
	GlaxoSmithKline, LLC.	$633,547
ACP	Gilead Sciences	$100,678
	AbbVie, Inc.	$75,724
	Boehringer Ingelheim	$60,588
APA	Pfizer	$216,008
	Sunovion Pharmaceuticals Inc.	$33,189
	Shire	$28,917

Note: payments from companies with the same name were aggregated.

Table 2 Guideline mentions of overdiagnosis, overuse, or related issues

Association	Guideline	Explicit Mention	Implicit Mention	Recommendation Mention
ACC	AF	No	No	No
	Cholesterol	No	Yes	No
	Hypertension	No	No	No
ACP	Back Pain	Yes	Yes	Yes
	Hypertension	No	No	No
	Diabetes	Yes	Yes	Yes
ACR	Ankylosing Spondylitis	Yes	No	Yes

	Psoriatic Arthritis	No	No	No
	Glucocorticoid Induced Osteoporosis	No	No	No
APA	Review	No	No	No
	Substance Use	No	No	No
	Suicide Risk	No	No	No
ASCO	Breast cancer TAILORx	No	No	No
	Human Epidermal Growth Factor	No	Yes	Yes
	Non–Small-Cell Lung Cancer	No	No	No
ATS	Management COPD	No	No	No
	Prevention COPD	No	No	No
	Severe Asthma	No	Yes	No
ES	Diabetes Older Adults	No	Yes	Yes
	Insulin & Monitoring	No	No	No
	Diabetes & Pregnancy	Yes	No	No
IDSA	Tonsillectomy	Yes	Yes	Yes
	Clostridium Difficile	Yes	Yes	Yes
	Ventriculitis & Meningitis	No	No	No
NASS	Adult isthmic spondylolisthesis	No	No	No
	Degenerative lumbar spondylolisthesis	No	No	No
	Antibiotics in Spine Surgery	No	No	No
OTA (only 1 available)	Trauma	No	No	No

Note for assessing guidelines: Explicit mentions include but are not limited to mention of words: Overdiagnosis , overuse, overtesting, overtreatment, unnecessary care, low value care, and related conjugations and synonyms. Implicit mentions could include mention of need to avoid unnecessary care. A passing reference to a finding from a specific trial relating to the safety of an intervention, or a recommendation not to use it because the evidence suggests it is unsafe or useless, is not considered an explicit or implicit mention of overdiagnosis or overuse.

List of Guidelines:

ACC:

January CT, Wann LS, Calkins H, et al. 2019 AHA/ACC/HRS focused update of the 2014 AHA/ACC/HRS guideline for the management of patients with atrial fibrillation: a report of the American College of Cardiology/American Heart Association Task Force on Clinical Practice Guidelines and the Heart Rhythm Society. Circulation. 2019;140:e125–e151.

Grundy SM, Stone NJ, Bailey AL, et al. 2018 AHA/ACC/AACVPR/AAPA/ABC/ACPM/ADA/AGS/APhA/ASPC/NLA/PCNA guideline on the management of blood cholesterol: executive summary: a report of the American College of Cardiology/American Heart Association Task Force on Clinical Practice Guidelines. Circulation. 2019;139:e1046–e1081.

Whelton PK, Carey RM, Aronow WS, et al. 2017 ACC/AHA/AAPA/ABC/ACPM/AGS/APhA/ASH/ASPC/NMA/PCNA Guideline for the Prevention, Detection, Evaluation, and Management of High Blood Pressure in Adults: Executive Summary: A Report of the American College of Cardiology/American Heart Association Task Force on Clinical Practice Guidelines. Hypertension. 2018;71:1269-1324.

ACP:

Qaseem A, Wilt T, McLean RM, et al. Noninvasive Treatments for Acute, Subacute, and Chronic Low Back Pain: A Clinical Practice Guideline From the American College of Physicians. Ann Intern Med. 2017;166(7):514-530.

Qaseem A, Wilt T, Rich R, et al. Pharmacologic Treatment of Hypertension in Adults Aged 60 Years or Older to Higher Versus Lower Blood Pressure Targets: A Clinical Practice Guideline From the American College of Physicians and the American Academy of Family Physicians. Ann Intern Med. 2017;166(6):430-437.

Qaseem A, Wilt T, Kansagara D, et al. Hemoglobin A1c Targets for Glycemic Control With Pharmacologic Therapy for Nonpregnant Adults With Type 2 Diabetes Mellitus: A Guidance Statement Update From the American College of Physicians. Ann Intern Med. 2018;168:569-576

ACR:

Ward MM, Deodhar A, Gensler LS, et al. 2019 Update of the American College of Rheumatology/Spondylitis Association of America/Spondyloarthritis Research and Treatment Network Recommendations for the Treatment of Ankylosing Spondylitis and Nonradiographic Axial Spondyloarthritis. Arthritis & Rheumatology. 2019, pp 1–15

Singh JA, Guyatt G, Ogdie A, et al. 2018 American College of Rheumatology/National Psoriasis Foundation Guideline for the Treatment of Psoriatic Arthritis. Arthritis & Rheumatology Vol. 71, No. 1, January 2019, pp 5–32

Buckley L, Guyatt G, Fink HA, et al. 2017 American College of Rheumatology Guideline for the Prevention and Treatment of Glucocorticoid-Induced Osteoporosis. Arthritis and Rheumatology. Vol. 69, No. 8, August 2017, pp 1521–1537

APA:

APA Work Group on Psychiatric Evaluation. The American Psychiatric Association Practice Guidelines for the Psychiatric Evaluation of Adults, Third Edition: Available at https://www.appi.org/products/practice-guidelines.

- GUIDELINE I. Review of Psychiatric Symptoms, Trauma History, and Psychiatric Treatment History

- GUIDELINE II. Substance Use Assessment
- GUIDELINE III. Assessment of Suicide Risk

ASCO:

Andre F, Ismaila N, Henry NL, et al. Use of Biomarkers to Guide Decisions on Adjuvant Systemic Therapy for Women With Early-Stage Invasive Breast Cancer: ASCO Clinical Practice Guideline Update—Integration of Results From TAILORx. J Clin Oncol 2019; 37:1956-1964.

Wolff AC, Hammond EH, Allison KH, et al. Human Epidermal Growth Factor Receptor 2 Testing in Breast Cancer: American Society of Clinical Oncology/ College of American Pathologists Clinical Practice Guideline Focused Update. J Clin Oncol 2018;36:2105-2122

Hanna N, Johnson D, Temin S, et al. Systemic Therapy for Stage IV Non–Small-Cell Lung Cancer: American Society of Clinical Oncology Clinical Practice Guideline Update. J Clin Oncol 2017;35:3484-3515.

ATS:

Wedzicha JA, Miravitlles M, Hurst JR, et al. Management of COPD exacerbations: a European Respiratory Society/American Thoracic Society guideline. Eur Respir J. 2017 Mar 15;49(3). pii: 1600791.

Wedzicha JA, Calverley PMA, Albert RK, et al. Prevention of COPD exacerbations: a European Respiratory Society/American Thoracic Society guideline. Eur Respir J. 2017 Sep 9;50(3). pii: 1602265

Chung KF, Wenzel SE, Brozek JL et al. International ERS/ATS guidelines on definition, evaluation and treatment of severe asthma. Eur Respir J 2014; 43: 343-373.

ES:

LeRoith D, Biessels GJ, Braithwaite SS, et al. Treatment of Diabetes in Older Adults: An Endocrine Society Clinical Practice Guideline. J Clin Endocrinol Metab 104: 1520–1574, 2019

Peters AL, Ahmann AJ, Battelino T, et al. Diabetes Technology—Continuous Subcutaneous Insulin Infusion Therapy and Continuous Glucose Monitoring in Adults: An Endocrine Society Clinical Practice Guideline. J Clin Endocrinol Metab 101: 3922–3937, 2016

Blumer I, Hadar E, Hadden DR, et al. Diabetes and Pregnancy: An Endocrine Society Clinical Practice Guideline. J Clin Endocrinol Metab 98: 4227–4249, 2013

IDSA:

Mitchell RB, Archer SM, Ishman SL, et al. Clinical Practice Guideline: Tonsillectomy in Children (Update)—Executive Summary. Otolaryngology–Head and Neck Surgery. 2019, Vol. 160(2) 187–205

Mc Donald LC, Gerding DN, Johnson S, et al. Clinical Practice Guidelines for Clostridium difficile Infection in Adults and Children: 2017 Update by the Infectious Diseases Society of America (IDSA) and Society for Healthcare Epidemiology of America (SHEA). Clinical Infectious Diseases. 2018;66(7):e1–e48

Tunkel AR, Hasbun R, Bhimraj A, et al. 2017 Infectious Diseases Society of America's Clinical Practice Guidelines for Healthcare-Associated Ventriculitis and Meningitis* Clinical Infectious Diseases. 2017;64(6):e34-e65

NASS:

Kreiner DS, Baisden J, Mazanec DJ, et al. Guideline summary review: an evidence-based clinical guideline for the diagnosis and treatment of adult isthmic spondylolisthesis. Spine J. 2016 Dec;16(12):1478-1485

Matz PG, Meagher RJ, Lamer T, et al. Guideline summary review: an evidence-based clinical guideline for the diagnosis and treatment of degenerative lumbar spondylolisthesis Spine J. 2016 Mar;16(3):439-48.

Shaffer WO. Baisden JL, Fernand R, et al. An evidence-based clinical guideline for antibiotic prophylaxis in spine surgery Spine J. 2013 Oct;13(10):1387-92

OTA :

ACS TQIP BEST PRACTICES IN THE MANAGEMENT OF ORTHOPAEDIC TRAUMA. American College of Surgeons, Orthopedic Trauma Association. Available at https://ota.org/sites/files/2018-04/TQIP-BPGs-in-the-Management-of-Orthopaedic-Traumafinal.pdf
