# Supplementary material for: Financial ties between leaders of influential US professional medical associations and industry: cross sectional study
Source: BMJ. 2020 May 27;369:m1505. doi: 10.1136/bmj.m1505 (PMC7251422; doi:10.1136/bmj.m1505)
Supplement: Supplementary file 2 — Supplementary information: study protocol [file moyr054932.ww1.pdf]

Research Project Description for use with HREA

BOND UNIVERSITY

Title and Resources

Title

Include the title and version number used on the HREA application

RM03089 : Financial ties between United States professional association leadership and industry: a cross sectional study

Resources

Describe the resources necessary to conduct the project including financial support

Resources will come from Institute for Evidence-Based Healthcare, at Bond University. Resources needed will be a modest mount of RA time, potentially 50-80 hours.

Background to the Project

Provide a brief plain language theoretical overview and project rationale.

There is a growing body of evidence detailing the nature and extent of financial entanglement between industry and health professionals, and the impacts of that entanglement in terms of threats to integrity of research, medical education and practice. (1-10) At the same time there is growing evidence and debate about the problem of too much medicine, and the role of commercial factors driving overdiagnosis, overuse and overmedicalization, across common diseases. (11-15)

Against a backdrop of growing concern about overdiagnosis, a cross sectional study of US based guideline panels which modify common disease definitions - often undertaken in connection with professional associations - found panels often expand definitions, and 75% of panel members had multiple ties to pharmaceutical companies with an interest in guideline deliberations.(14) In 2019, there are calls for major reforms to the way diseases are defined – to address overdiagnosis – which include much greater independence from vested interests, including industry. (15)

While there is on-going debate about the closeness between professional associations and industry, (16-20) few studies have looked at the relationships between industry and professional associations which oversee influential guidelines, fund research, and run medical education. (18,20) To our knowledge no studies have undertaken a cross sectional analysis of the most influential professional associations, using the US Open Payments database, which since 2013 has disclosed – by US law – every payment to every US based medical doctor from drug and device makers.(21)

In this study we aim to analyse any financial relationships between the medical leadership of leading professional associations in the United States, US, and drug or device makers. For the purpose of our study a leading association will be identified within each of the ten most costly disease/condition areas by a group of relevant US based professional peers. A secondary aim is to assess recent guidelines from these associations, to determine whether concerns relating to overuse or overdiagnosis are mentioned.

Project team includes: Assistnt Professor Dr Ray Moynihan, Dr Loai Albaqouni, Professor Lisa Bero, Professor Joel Lexchin, Associate Professor Adam Dunn, Mr Conrad Nangla.

Describe the research questions, aims, objectives and hypothesis

Questions: (i) What is the nature and extent of financial relationships between the medical leadership of leading US professional associations, and drug and device makers. (ii) To what extent are guidelines produced by these organisations including mention of overuse or overdiagnosis.

Aims and objectives: We aim to conduct a cross-sectional study of leading professional associations active within common disease areas, and examine any ties between their medical leadership and drug and device makers, and any mention of overuse or overdiagnosis in guidelines produced by these organisations.

Hypothesis: We hypothesise that there will be evidence of financial ties between medical leadership of professional associations and industry, with limited attention in association guidelines to issues of overuse and overdiagnosis.

Methods:

Identifying disease categories: We will use the most recent US Agency for Healthcare Research and Quality data (2015) to identify the top ten most costly diseases in the US. (22) The most costly disease areas represent the areas of large disease burden, as well as areas where there is potential concern about industry influence, and the potential existence of overuse and overdiagnosis.

Identifying professional associations: For each disease area, we will identify the leading professional association, (which produces guidelines) through recommendations from (for each disease category) three US-based expert peers, per category.

Identifying organizational leadership: Using published materials, including organization websites and other documents where necessary such as annual reports, we will identify the leadership of each identified organization. Leadership to be included will be members of the organisations overarching body – such as a Board, or Council. We will include members for current year, eg 2018-2019, and two years previous. The justification for three years, arises from the date of commencement of the Open Payments system. In line with WHO guidance (23) we are interested in any financial relationships in the current year, and 4 years previous to membership, and one year following membership, of the leadership group – where this is available in the publicly accessible US CMS Open Payments database. Non-board staff will be excluded. For US based medical leadership, eg those with an MD and eligible to be included in the database, a search will be conducted of the US CMS Open Payments system database, to identify if each leader is present in the database, for any year since 2013.

Two authors will independently identify organizational leadership and within that group the US based medical leadership, and whether or not leaders appear in Open Payments database. This will involve the use of biographical material to ensure a correct match between the association leader and the person identified in Open Payments. Discrepancies will be resolved by discussion, with disagreements resolved by a third author. We will also note whether the association includes disclosure information in the current listings of their leadership.

Identifying and extracting details from Open Payments: Once there is certainty that the leader matches the entry in Open Payments, one author will extract data on the identified medical leaders, for each and every year available. This will include the nature and extent of payments, using categories within the Open Payments database. General categories include General Payments, Research and other. General payments has multiple sub-categories, including for example, consultancies, food and beverage, travel etc. These categories will be established in an extraction form. Publicly available data on the details of payments for each leader will also be downloaded, including the names of the companies making the payments. A second author will double check a random sample of at least 10% of the leaders' extractions for accurary.

Identifying guidelines for assessment: Two authors will independently identify guidelines from each identified professional association, related to the relevant disease category, and select the guidline with the highest combination of downloads and citations for each of the three previous years, eg 2016, 2017, 2018. (2019 guidelines will have few citations, as at date of this application). Discrepancies will be resolved by discussion, and a third author in cases of disagreement. Method for assessing mention of overdiagnosis and overuse will draw on methods previously used by authors. (13)

Analysis: We will investigate the following outcomes; (primary) proportion of all leadership (including non-MD and non- US resident) with ties in current year of serving as leader, previous 4 years and following year – contingent on data availability; proportion of medical US based leadership with ties, overall, and within each organisations; proportion of organisations with no leaders with ties; extent/nature of ties among those with ties, including for example total dollar amounts overall, and within categories, and amounts per organization and per leader; the leading three companies, with most ties in terms of dollar amounts, overall, and per category, per organisation, per leader; (secondary) proportion of top three guidelines (per organization for the target condition) mentioning issues related to overuse and overdiagnosis; any changes in extent or nature of ties over time period of the study, according to any of the metrics of analysis.

Describe the expected outcomes and impacts of the research

We expect the outcomes to throw more light on the financial relationships between the leadership of influential professional associations and industry, to help inform current debates about conflicts of interest and moves towards more independence from commercial influences.

Provide a brief list of your key references- DRAFT

1. Lo B , Field MJ *Conflict of interest in medical research, education, and practice. Editors: Institute of Medicine (US) Committee on conflict of interest in medical research, education, and practice*. Washington (DC): National Academies Press (US), 2009
2. Institute of Medicine. *Clinical Practice Guidelines We Can Trust, Report Brief*. Washington, DC: The National Academies Press, 2011
3. Moynihan R, MacDonald H, Heneghan C, et al. Commercial Interests, transparency, and independence: a call for submissions. *BMJ* 2019;365:l1706
4. Lundh A , Lexchin J , Mintzes B , et al Industry sponsorship and research outcome. *Cochrane database of systematic reviews*. First published 16 February 2017.
5. Want T, McCoy P, Murad, M et al. Association between industry affiliation and position on cardiovascular risk with rosiglitazone: cross sectional systematic review. *BMJ* 2010;340:c1344

6. Nusrat S, Syed T, Nusrat S, et al. Assessment of Pharmaceutical Company and Device Manufacturer Payments to Gastroenterologists and Their Participation in Clinical Practice Guideline Panels. *JAMA Netw Open*. 2018 Dec; 1(8): e186343
7. Haque W, Minhajuddin A, Gupta A, et al. Conflicts of interest of editors of medical journals. *PLoS ONE* 2018 13(5): e0197141. https://doi.org/10.1371/journal.pone.0197141
8. Liu JJ, Bell CM, Matelski JJ, et al. Payments by US pharmaceutical and medical device manufacturers to US medical journal editors: retrospective observational study. *BMJ.* 2017 Oct 26;359:j4619. doi: 10.1136/bmj.j4619
9. Rothman D, McDonald W, Berkowitz C, et al. Professional medical associations and their relationships with industry: a proposal for controlling conflict of interest. *JAMA.* 2009;301(13):1367-1372
10. Dunn AG , Coiera E , Mandl KD , *et al* Conflict of interest disclosure in biomedical research: a review of current practices, biases, and the role of public registries in improving transparency. *Res Integr Peer Rev* 2019;1:1. Epub 2016 May 3.doi:10.1186/s41073-016-0006-7
11. Jatoi I, Sah S. Clinical practice guidelines and the overuse of health care services: need for reform. *CMAJ* March 18, 2019 191 (11) E297-E298

12. Glasiou P, Moynihan R, Richards T, et al.Too Much Medicine Too Little Care. *BMJ* 2013;347:f4247
13. Pathirana T, Clark J, Moynihan R Mapping the drivers of overdiagnosis to potential solutions. *BMJ* 2017;358:j3879.doi:10.1136/bmj.j3879 pmid:28814436
14. Moynihan RN , Cooke GP , Doust JA , *et al* Expanding disease definitions in guidelines and expert panel ties to industry: a cross-sectional study of common conditions in the United States. *PLoS Med* 2013;10:e1001500
15. Moynihan R, Brodersen J, Heath I, et al. Reforming disease definitions: a new primary care led, people-centred approach. *BMJ Evidence-Based Medicine* Epub ahead of print,April 2019.
16. Smith CD, Buyske J, Talamini MA. Industry support and professional medical associations.

JAMA. 2009 Aug 19;302(7):738-9; author reply 739. doi: 10.1001/jama.2009.1195. No abstract available.

17. Rothman DJ. Professional Medical Associations and Divestiture from Industry: An Ethical Imperative for Pain Society Leadership. *Pain Med*. 2016 Feb;17(2):218-9. No abstract available
18. Fabbri A, Gregoraci G, Tedesco D, et al. Conflict of interest between professional medical societies and industry: a cross-sectional study of Italian medical societies' websites. *BMJ Open*. 2016 Jun 1;6(6):e011124.
19. Schofferman JA, Eskay-Auerbach ML, Sawyer LS, et al. Conflict of interest and professional medical associations: the North American Spine Society experience. *Spine J.* 2013 Aug;13(8):974-9.
20. Saito H, Ozaki A , Kobayashi, Y et al. Pharmaceutical Company Payments to Executive Board Members of Professional Medical Associations in Japan *JAMA Int. Med*. Research Letter E2 Published Online February 4, 2019 .
21. Centres for Medicare & Medicaid Services, Open Payments database. https://www.cms.gov/openpayments/ (accessed 12 Apr 2018).

22. Agency for Healthcare Research and Quality. Total expenditures in millions by condition, United States, 2015. Medical Expenditure Panel Survey. Generated interactively: Wed Mar 27 2019. https://meps.ahrq.gov/mepstrends/hc_cond/#plot-tab
23. World Health Organization. *WHO Conflict of Interest Guidelines*: declaration of interests for WHO experts, 2010. http://keionline.org/node/1062. (accessed 12 Apr 2018).doi:10.1016/j.jbi.2008.08.010

Project Design

Describe the Rationale for your choice of research methods as specified in the HREA. How do these support the research objectives of your research project?

A cross-sectional study using publicly available data is the ideal method to learn of the nature and extent of financial relationships between the leadership of influential professional associations and medical industries.

Provide a description of the participants planned for your study. How many you will need? What are your inclusion/exclusion criteria? How will participants be recruited? What is your estimate and rationale of sample size?

As per material in the section above "Describe the research questions, aims, objectives and hypothesis" - there will be no participants. All data will be easily publicly available and accessible, from the US Open Payments database. We will not be identifying a sample of leaders, we will identify all leadership for the ten identified organisations, over the three year period.

Leadership to be included will be members of the organisations overarching body – such as a Board, or Council. We will include members for current year, eg 2018-2019, and two years previous. The justification for three years, arises from the date of commencement of the Open Payments system. In line with WHO guidance (23) we are interested in any financial relationships in the current year, and 4 years previous to membership, and one year following membership, of the leadership group – where this is available in the publicly accessible US CMS Open Payments database. Non-board staff will be excluded.

What instruments / materials are you using? If possible provide validity and reliability of instruments or address the validity of the method.

We will use publicly available materials, such as the AHRQ list of the ten most costly diseases in the US, professional organisation websites, and the US Open Payments database.

Describe your procedure. What will participants be asked to do? How much time is required of paricipants at a test session and in total? Will there be any follow-up?

There are no participants.

How will you handle the withdrawal/loss of any participants from your study?

There are no participants.

Describe your data analysis. What are your measures? How will data be coded? Include any matching and sampling strategies, data linkages, strategies for accounting for potential bias, confounding factors and missing information and statistical power calculation.

Analysis: We will investigate and report the following outcomes; (primary) proportion of all leadership (including non-MD and non- US resident) with ties in current year of serving as leader, previous 4 years and following year – contingent on data availability; proportion of medical US based leadership with ties, overall, and within each organisations; proportion of organisations with no leaders with ties; extent/nature of ties among those with ties, including for example total dollar amounts overall, and within categories, and amounts per organization and per leader; the leading three companies, with most ties in terms of dollar amounts, overall, and per category, per organisation, per leader; (secondary) proportion of top three guidelines (per organization for the target condition) mentioning issues related to overuse and overdiagnosis; any changes in extent or nature of ties over time period of the study, according to any of the metrics of analysis.

For research involving an investigational drug or device as part of a clinical trial: What is/are the drug(s) and/or device(s):

- Approved name
- Trade name (if any)
- Manufacturer
- Supplier of drug/device (e.g. manufacturer/pharmacy)
- Approved therapeutic indication, dosage/duration in Australia
- Believed mode of action
- Dosage regimen
- Mode of excretion
- Known adverse events
- Known contra-indications or warnings
- If arrangements have been made for the Pharmacy Department to receive or dispense the drugs involved in this project, explain how the drugs will be received and dispensed for the purposes of the research project.

n/a

Closure and Dissemination

Ethics approval covers not only the collection of data but also issues such as dissemination of data, researchers' duty of care for participants after data collection, and responsibilities to the institution, professional bodies and research partners/sponsors. This is the closure phase of research.

How do you plan to disseminate the results of your research, including to participants and stakeholders? Include any publications that are planned as a result of this research.

We plan to report the findings of this study in a peer-reviewed publication, and in scientific conferences, in Australia and internationally.

Describe the project closure processes and plans for any follow-up research.

We will finalise the project once data collection, analysis and publication and dissemination are complete. The results will inform on-going research into conflicts of interest, research integrity and overdiagnosis.

Data Management and Sharing

What will be the format of your research outputs(e.g. survey data,photographs, publications, csv files, etc)? Will they require physical and/or digital storage for the mandatory data retention period? Where will the data be stored? Will the data be automatically or manually backed up? What arrangements have been made to archive the data? What will be done at the end of the mandatory retention period? Will you include measurements from experiments, surveys, photographs, or publications?

Research outputs will be simple word documents and Excel spreadsheets. Data will be stored for the mandatory data retention period of 5 years, in the Bond University Drive, which is backed up automatically by Bond University IT services

List any relevant policies and/or legislation that affect your data. Does any of your data contain personal or commercially valuable information?

No. All the data we are analysing will come from the publicly accessible Open Payments system website in the US.

Who will own the data? Is there any reason why your data should not be made publicly available? If so, provide a rationale. Who would be interested in your data?

The data generated through this study will be owned and retained by Bond University. A summary of de-identified results will be made publicly available. Those interested in using our data would be other researchers, although a much more complete dataset of the same data is freely and publicly available via the Open Payments database.

Describe how your data will be shared with other researchers and through what channels. What provisions have you made to store and share your data via a publicly accessible repository? Will there be an embargo period? If the data cannot be open, will there be negotiated or controlled access to your data?

Beyond the publication of results in a peer-reviewed journal, and potentially dissemination at conferences, a summary of de-identified results will be available through the Bond University publicly accessible repository.

How will you ensure the security and integrity of the data and handling of any confidential or sensitive data?

The de-identified results/data made publicly available will not include any confidential or sensitive data.

It is a condition of ethical approval that all data created as part of research projects is stored on approved Bond University network storage facilities for a minimum retention period of 5 years. Some data should be retained permanently. Refer to the University Sector Retention and Disposal Schedule: Research Data for guidance. If data needs to be stored elsewhere, please detail the reason for this and outline security and backup procedures that will be maintained.

There is no need for the data to be stored elsewhere.
