# Supplementary material for: Financial ties between leaders of influential US professional medical associations and industry: cross sectional study
Source: BMJ. 2020 May 27;369:m1505. doi: 10.1136/bmj.m1505 (PMC7251422; doi:10.1136/bmj.m1505)
Supplement: Supplementary file 3 — Supplementary information: email sent to expert peers [file moyr054932.ww2.pdf]

Email to peers.

Hello Dr …

I got your name from the Cochrane Infectious Disease group website https://cidg.cochrane.org/who-we-are . (Coincidentally I host a podcast produced by Cochrane Australia called The Recommended Dose)

I am also a researcher based in Australia, at Bond University, and just wanted to email you with a very quick question.

We are in the early stages of a study and are trying to identify the most influential/important professional organisations in the United States across 10 diseases/conditions – specifically we are aiming to identify one professional association for each of the top 10 most costly disease/condition categories, according to AHRQ data analysis.

One of the top 10 is "Infectious Diseases" which in AHRQ classification includes the conditions listed at the end of this email.

As someone with a knowledge of infectious diseases can you suggest the two most influential/important professional associations (eg College, Academy, Society, Association etc..?) in the area of infectious disease treatments in the US – ie organisations to which medical doctors/physicians/surgeons who treat this disease/condition area would belong, and which would be involved, among other activities, in producing guidelines.

I hope you don't mind me asking you this, and please feel free to ignore if you do not have time or inclination, and please let me know if the question is not clear enough.

Cheers and thanks

Dr Ray Moynihan (Signature below)

1 Tuberculosis

2 Septicemia (except in labor)

3 Bacterial infection; unspecified site

4 Mycoses

5 HIV infection

6 Hepatitis

7 Viral infection

8 Other infections; including parasitic

9 Sexually transmitted infections (not HIV or hepatitis)
